# Framework humanization enhances GM3(Neu5Gc)-targeting CAR-T cell function by reducing tonic signaling

**DOI:** 10.3389/fimmu.2025.1697732

**Published:** 2025-10-23

**Authors:** Jiaxin Tu, Xinyu Li, Yuge Zhu, Shance Li, Guanyu Zhang, You He, Chaoting Zhang, Zheming Lu

**Affiliations:** ^1^ Key Laboratory of Carcinogenesis and Translational Research (Ministry of Education/Beijing), Laboratory of Biochemistry and Molecular Biology, Peking University Cancer Hospital & Institute, Beijing, China; ^2^ Key Laboratory of Carcinogenesis and Translational Research (Ministry of Education/Beijing), Department of Thoracic Surgery II, Peking University Cancer Hospital & Institute, Beijing, China

**Keywords:** GM3(Neu5Gc) CAR-T cells, framework humanization, tonic signaling, car-t, solid tumor

## Abstract

GM3(Neu5Gc), a tumor-associated ganglioside absent in normal human tissues due to a CMP-N-acetylneuraminic acid hydroxylase (CMAH) mutation, is an attractive target for solid tumor immunotherapy. To advance the clinical potential of GM3(Neu5Gc)-targeted CAR-T cells, we systematically evaluated antibody humanization by comparing CARs based on the murine 14F7 antibody and its humanized variant 14F7hT. Within each scFv framework, we further assessed three hinge domains (CD8α, CD28, IgG4) to optimize CAR design. While hinge selection influenced *in vitro* cytotoxicity—favoring CD28—the humanized 14F7hT-based CARs consistently outperformed their murine counterparts *in vivo*. The optimized 14F7hT-CD28 CAR-T cells demonstrated superior expansion, persistence, tumor infiltration, and antitumor efficacy in xenograft models. To further characterize the basis of this *in vivo* advantage, we performed a repeated tumor stimulation assay mimicking chronic antigen exposure in solid tumors. In this setting, hGM3/CD28 CAR-T cells exhibited enhanced cytotoxicity, degranulation, and proliferation, supporting improved functional durability. Mechanistically, this was linked to reduced tonic signaling: CAR-Toner predicted a near-optimal signal strength for hGM3/CD28 (score 59) versus excessive signaling in mGM3/CD28 (score 65). This was confirmed experimentally by lower basal cytokine secretion and activation marker expression in cytokine- and antigen-free conditions. Despite preserving complementarity-determining regions, scFv humanization induced subtle structural changes that attenuated tonic signaling and enhanced CAR-T functionality. These findings underscore a critical, previously underappreciated role for antibody framework regions in modulating CAR signaling and therapeutic efficacy. Our study establishes 14F7hT-CD28 as a promising candidate for GM3(Neu5Gc)-positive tumors and highlights framework humanization as a key strategy to improve CAR-T cell performance.

## Introduction

Chimeric antigen receptor (CAR) T cell therapy has shown remarkable success in treating hematologic malignancies and is being actively investigated for the treatment of solid tumors (1–3). A key determinant of CAR-T efficacy and safety is the selection of an appropriate tumor-associated antigen that is highly expressed on cancer cells but minimally or not at all expressed on normal tissues (4, 5).

GM3(Neu5Gc), a variant of the ganglioside GM3 containing N-glycolylneuraminic acid, fulfills this criterion (6). Due to the inactivation of the CMP-N-acetylneuraminic acid hydroxylase (CMAH) gene, humans are unable to synthesize Neu5Gc, resulting in its absence in normal tissues (6). However, Neu5Gc can be incorporated into tumor cells from dietary sources or upregulated through aberrant metabolic pathways, leading to selective expression of GM3(Neu5Gc) in various human cancers, including ovarian, lung, and breast tumors, while remaining undetectable in healthy tissues (6–9). This tumor-restricted expression profile makes GM3(Neu5Gc) an attractive target for CAR-T cell therapy.

Two prior studies have reported the development of GM3(Neu5Gc)-targeting CAR-T cells using the murine monoclonal antibody 14F7 as the source of the single-chain variable fragment (scFv), demonstrating potent antitumor activity in preclinical models without detectable toxicity to normal tissues (10, 11). These studies employed different hinge domains—CD8α in one and CD28 in the other—but did not conduct a direct comparison, despite growing evidence that hinge selection can critically affect CAR-T cell flexibility, epitope accessibility, and signaling strength (12–14). A systematic evaluation of hinge domains is therefore important for optimizing CAR configuration, particularly for solid tumors. Moreover, both studies exclusively used murine-derived scFvs, which, although effective, may trigger immune responses that impair CAR-T cell persistence and therapeutic efficacy in clinical settings.

To address these limitations, we designed a panel of GM3(Neu5Gc)-targeting CAR constructs using both the original murine 14F7 and its humanized counterpart, 14F7hT (15). The humanized 14F7hT retains the antigen-binding complementarity-determining regions (CDRs) of 14F7 but substitutes the framework regions (FRs) with human sequences, thus preserving binding affinity while potentially reducing immunogenicity. Each scFv was paired with one of three widely used hinge domains—CD28, CD8α, or IgG4—and evaluated *in vitro* and *in vivo* to identify the optimal CAR configuration. Among these, the CD28 hinge consistently conferred the strongest antitumor activity.

Interestingly, while 14F7-CD28 and 14F7hT-CD28 CAR-T cells exhibited comparable *in vitro* function, including antigen specificity, cytokine secretion, and short-term cytotoxicity, the 14F7hT-CD28 CAR-T cells exhibited markedly enhanced *in vivo* expansion, persistence, and tumor control. This was associated with enhanced CAR-T cell expansion and infiltration into tumor tissues. Extended *in vitro* assays simulating repeated antigen exposure revealed that 14F7hT-CD28 CAR-T cells had superior proliferative capacity and sustained cytotoxicity over time compared to their murine counterparts.

Mechanistic investigations revealed that 14F7hT-CD28 CAR-T cells exhibited reduced tonic signaling—reflected by lower antigen-independent activation—despite preserving the same complementarity-determining regions (CDRs). This highlights that subtle changes introduced by framework humanization alone can modulate CAR structural dynamics and downstream signaling (16).

Taken together, our study not only identifies an optimized CAR construct targeting GM3(Neu5Gc) but also reveals an underappreciated role of scFv framework humanization in modulating CAR-T cell signaling and performance. These insights support the clinical potential of 14F7hT-CD28 CAR-T cells for the treatment of GM3(Neu5Gc)-positive tumors.

## Results

### CD28 hinge domain enhances activation and cytotoxic function of murine GM3(Neu5Gc)-specific CAR-T cells *in vitro*

To evaluate the antitumor efficacy of CAR-T cells targeting the tumor-associated ganglioside GM3(Neu5Gc), we first generated a stable GM3(Neu5Gc)-expressing SKOV3 ovarian cancer cell line (SKOV3-CMAH) via lentiviral transduction of the murine CMAH gene, which reconstitutes the biosynthetic pathway for Neu5Gc. Flow cytometric analysis confirmed robust and homogeneous expression of GM3(Neu5Gc) on SKOV3-CMAH cells, while parental SKOV3 cells remained negative, establishing a reliable antigen-positive target model (**Supplementary Figure S1**).

**Figure 1:**
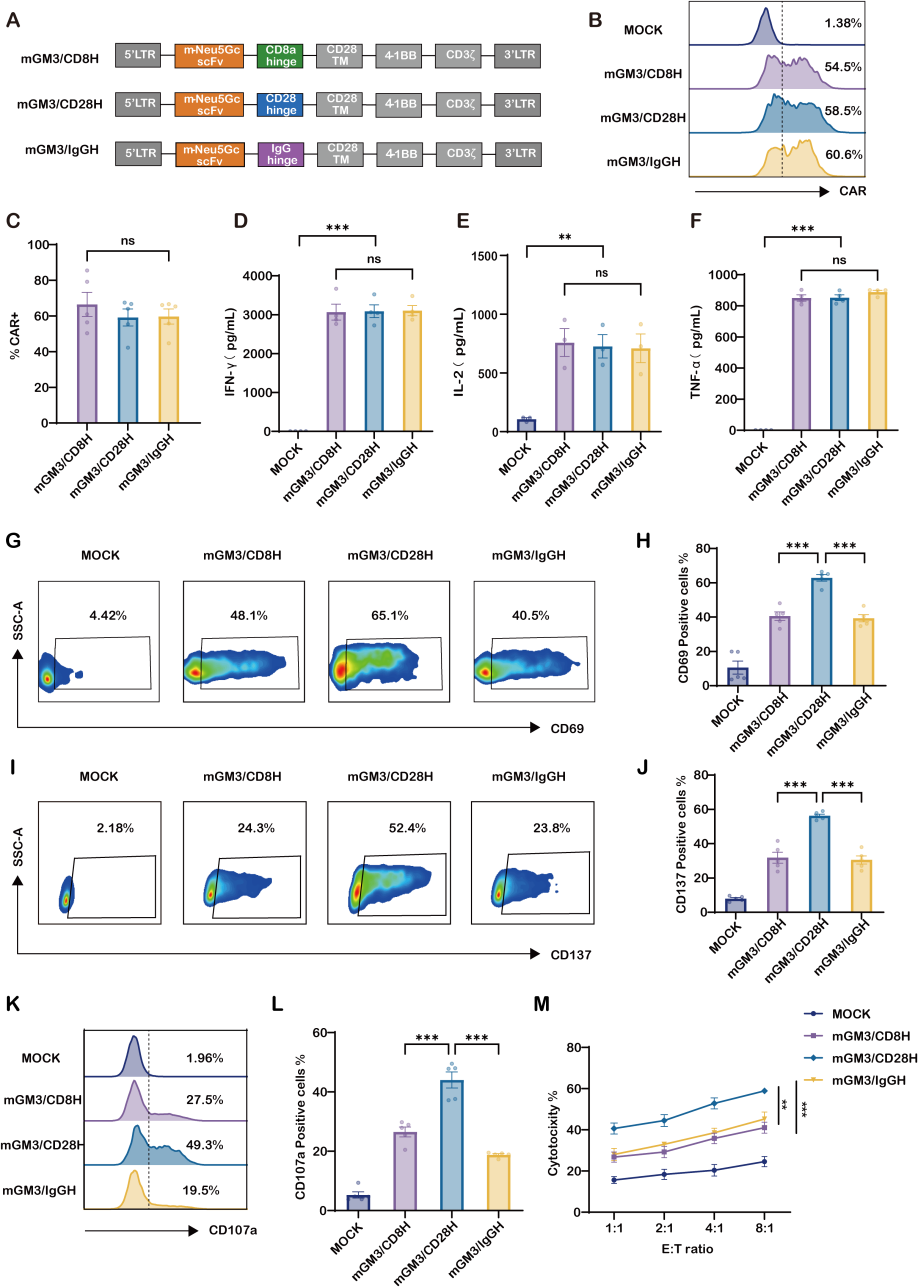
CD28 hinge enhances activation and cytotoxicity of murine GM3(Neu5Gc)–CAR-T cells *in vitro*. **(A)** Schematic of three second-generation CAR constructs targeting GM3(Neu5Gc), each comprising the murine 14F7 scFv, a hinge region (CD8α, CD28, or IgG4), CD28 transmembrane domain, 4-1BB costimulatory domain, and CD3ζ signaling domain. **(B)** Representative flow cytometry histograms showing CAR surface expression on primary human T cells 72 h after lentiviral transduction (mock, mGM3/CD8H, mGM3/CD28H, mGM3/IgGH). Dotted line indicates isotype control. **(C)** Quantification of CAR^+^ transduction efficiency (n = 5), showing comparable expression across constructs. **(D–F)** Cytokine production following 24 h co-culture with GM3(Neu5Gc)^+^ SKOV3-CMAH cells (E:T = 5:1): ELISA measurements of **(D)** IFN-γ (n = 4), **(E)** IL-2 (n = 3), and **(F)** TNF-α (n = 4). **(G–J)** Activation marker expression: representative plots for **(G)** CD69 and **(I)** CD137, with quantification of **(H)** CD69^+^ and **(J)** CD137^+^ cells (n = 5). **(K, L)** Degranulation assessed by CD107a mobilization upon antigen stimulation: **(K)** representative histograms and **(L)** percentage of CD107a^+^ cells (n = 5). **(M)** Tumor cell lysis assessed by flow cytometry–based cytotoxicity assay at increasing E:T ratios (1:1 to 8:1) over 12 h (n = 5). ** indicates p < 0.01; *** indicates p < 0.001; ns, not significant.

We next engineered three CAR constructs containing the murine anti-GM3 scFv (14F7), differing only in their hinge regions: CD8α (mGM3/CD8H), CD28 (mGM3/CD28H), and IgG4 (mGM3/IgGH) (**Figure 1A**). Comparable CAR expression on T cells was verified across constructs (**Figures 1B, C**). Upon co-culture with SKOV3-CMAH cells, all three CAR-T cell variants produced similar levels of key effector cytokines IFN-γ, IL-2, and TNF-α (**Figures 1D–F**). However, mGM3/CD28H CAR-T cells showed significantly increased expression of the activation marker CD69 and co-stimulatory receptor CD137, suggesting superior activation potential (**Figures 1G–J**). In line with this, CD107a degranulation assays revealed enhanced cytotoxic granule release in mGM3/CD28H cells (**Figures 1K, L**), and cytotoxicity assays demonstrated consistently higher tumor-killing efficiency across all tested effector-to-target (E:T) ratios (**Figure 1M**). These results support the CD28 hinge as a functionally superior structural element that augments activation and effector functions of GM3-specific CAR-T cells.

### CD28 hinge similarly enhances the activity of humanized GM3-CAR-T cells *in vitro*

**Figure 2:**
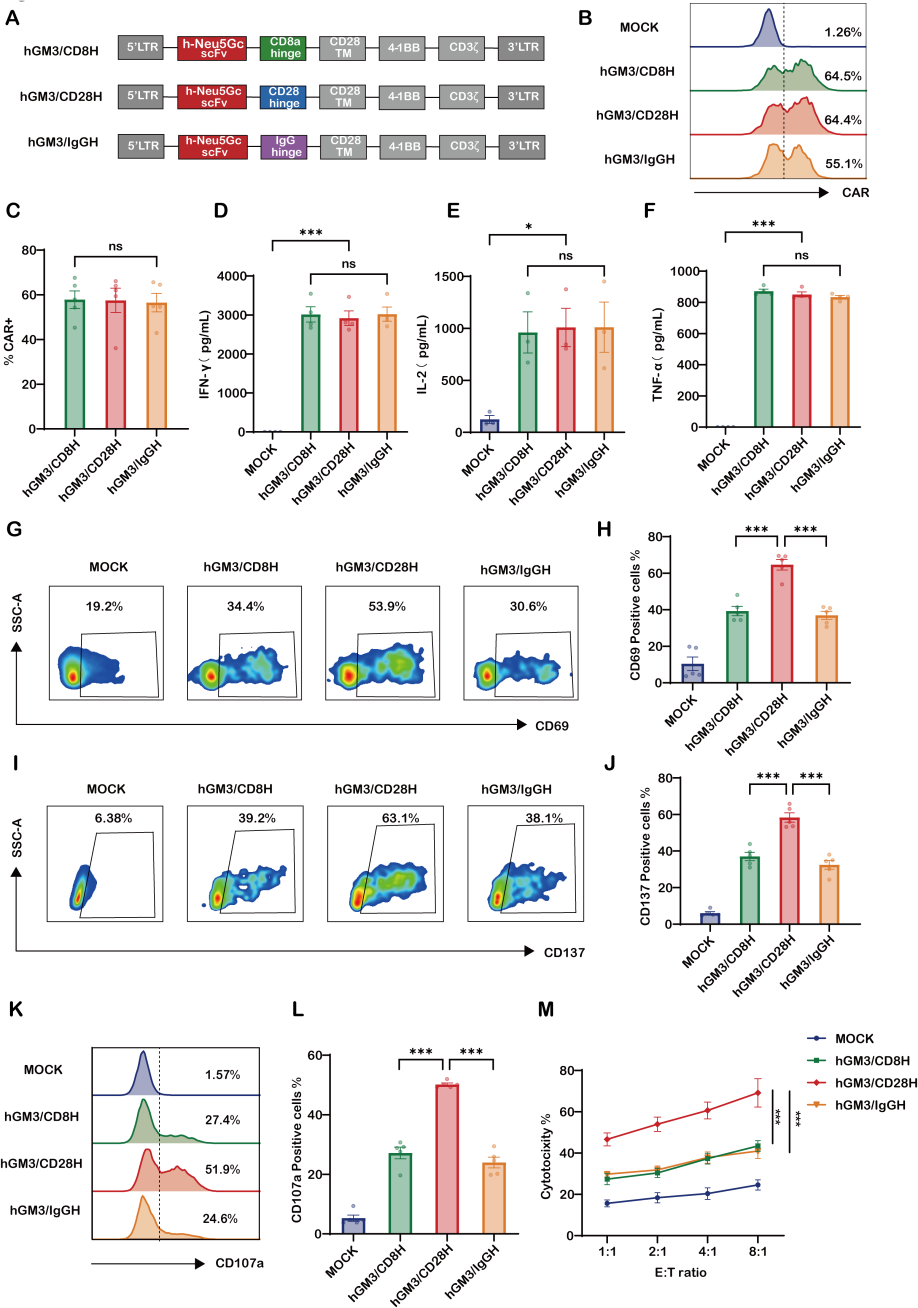
CD28 hinge enhances activation and cytotoxicity of humanized GM3(Neu5Gc)–CAR-T cells *in vitro*. **(A)** Schematic of CAR constructs containing the humanized 14F7hT scFv with CD8α, CD28, or IgG4 hinge, CD28 transmembrane domain, 4-1BB, and CD3ζ. **(B)** Flow cytometry histograms showing CAR expression 72 h post-transduction (mock, hGM3/CD8H, hGM3/CD28H, hGM3/IgGH). **(C)** Quantification of CAR^+^ cells (n = 5), demonstrating similar transduction across groups. **(D–F)** Cytokine secretion following 24 h co-culture with SKOV3-CMAH (E:T = 5:1): ELISA for **(D)** IFN-γ (n = 4), **(E)** IL-2 (n = 3), **(F)** TNF-α (n = 4). **(G–J)** Activation marker expression: representative plots for **(G)** CD69 and **(I)** CD137, with quantification of **(H)** CD69^+^ and **(J)** CD137^+^ cells (n = 5). **(K, L)** CD107a degranulation assay: **(K)** representative plots and **(L)** quantification (n = 5). **(M)** Cytotoxicity assay over 12 h with increasing E:T ratios (1:1 to 8:1) (n = 5). * indicates p < 0.05; *** indicates p < 0.001; ns, not significant.

To mitigate immunogenicity associated with murine scFvs and facilitate clinical translation, we humanized the 14F7 scFv (14F7hT) by grafting murine CDRs onto a human antibody framework (**Supplementary Figure S2**), and generated analogous CD8H, CD28H, and IgGH constructs using this humanized scFv (**Figure 2A**). All humanized CAR-T cell variants exhibited comparable CAR surface expression levels (**Figures 2B, C**).

Upon stimulation with SKOV3-CMAH cells, cytokine secretion profiles were similar among the three constructs (**Figures 2D–F**). However, hGM3/CD28H CAR-T cells showed significantly higher frequencies of CD69+ and CD137+ cells, indicating more potent early activation and co-stimulatory signaling (**Figures 2G–J**). Additionally, CD107a degranulation and cytotoxicity assays demonstrated that hGM3/CD28H CAR-T cells exerted superior tumor cell killing relative to CD8H and IgGH variants (**Figures 2K–M**). Together, these findings affirm that the functional benefit conferred by the CD28 hinge is preserved in the humanized CAR-T format, positioning hGM3/CD28H as the lead candidate for further development.

### Murine and humanized CD28H-based GM3-CAR-T cells exhibit comparable *in vitro* potency

We next directly compared mGM3/CD28H and hGM3/CD28H CAR-T cells to determine whether humanization impacted *in vitro* functional properties. Both constructs showed equivalent CAR expression on primary T cells (**Supplementary Figures S3A–C**). After co-culture with SKOV3-CMAH cells, the two CAR-T cell types secreted comparable levels of IFN-γ, IL-2, and TNF-α (**Supplementary Figures S3D–F**), and expressed similar levels of CD69 and CD137 (**Supplementary Figures S3G–J**), reflecting equivalent activation profiles.

CD107a degranulation assays showed no significant differences in granule release (**Supplementary Figures S3K, L**), and cytotoxicity assays confirmed that both constructs mediated potent and comparable tumor cell lysis across multiple E:T ratios (**Supplementary Figure S3M**). These data indicate that humanization of the scFv does not compromise CAR expression, antigen recognition, or effector function *in vitro*, supporting further *in vivo* testing of both formats.

### Humanized CD28H-based GM3-CAR-T cells display superior *in vivo* antitumor efficacy and tumor infiltration

**Figure 3:**
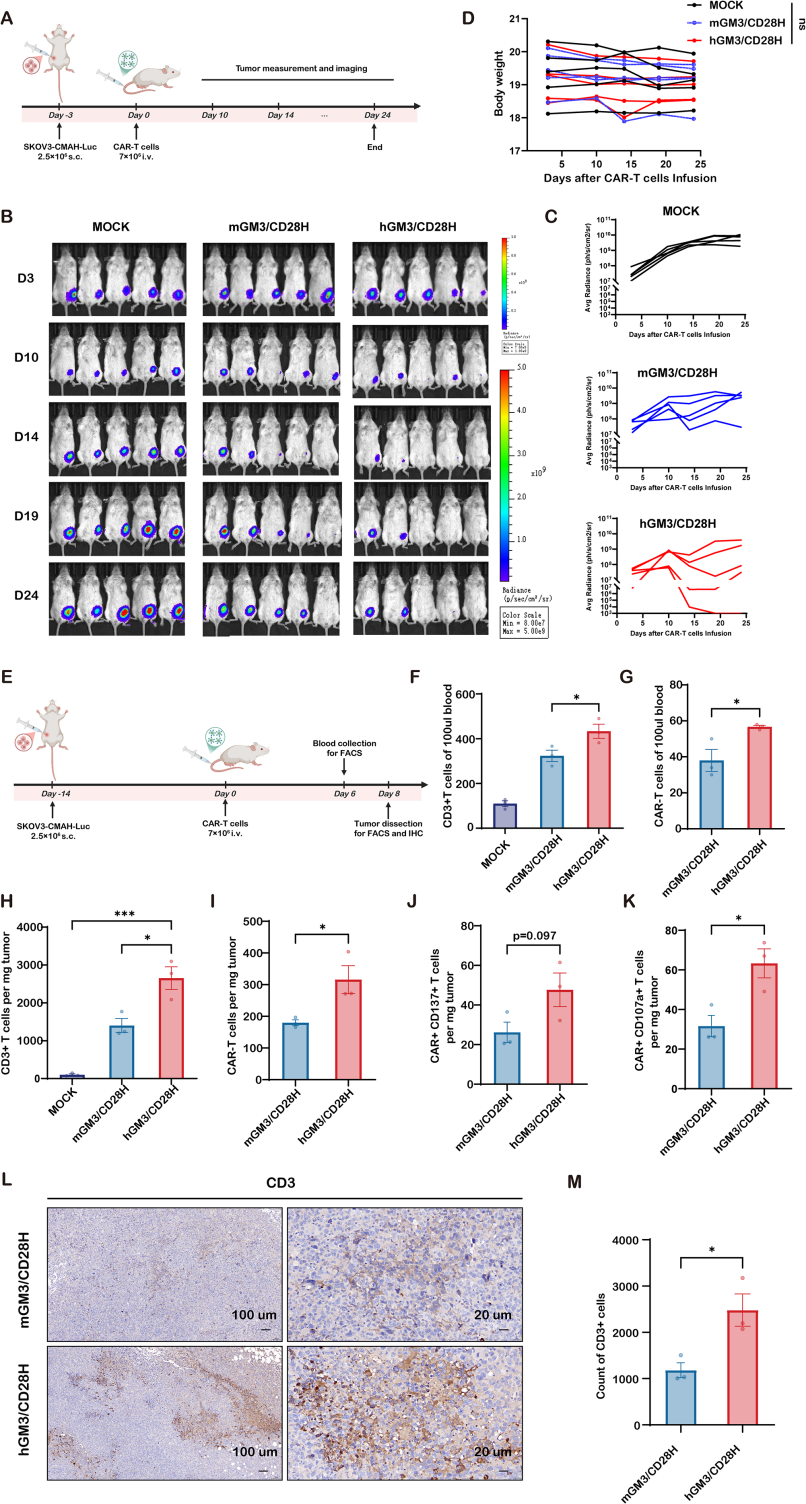
*In vivo* antitumor efficacy of murine and humanized CD28-hinged GM3 CAR-T cells. **(A)** Experimental timeline: SKOV3-Luc cells (2.5 × 10^6^) were injected subcutaneously on day –3; mice received mock, mGM3/CD28H, or hGM3/CD28H CAR-T cells (7 × 10^6^) on day 0. Tumor growth was monitored by bioluminescence imaging (BLI); body weight tracked throughout. **(B)** Representative BLI images at days 3, 10, 14, 19, and 24, pseudocolor scale indicates photon flux. **(C)** BLI kinetics for individual mice (n = 5/group). **(D)** Body weight monitoring. **(E)** Sampling schematic for flow cytometry and IHC on day 8 in a second cohort. **(F, G)** Frequency of **(F)** CD3^+^ and **(G)** CAR^+^ cells in peripheral blood (n = 3). **(H–K)** Intratumoral immune profiling: absolute numbers per mg tumor of **(H)** CD3^+^ T cells, **(I)** CAR-T cells, **(J)** CD137^+^ CAR-T cells, and **(K)** CD107a^+^ CAR-T cells (n = 3). **(L, M)** IHC for CD3^+^ T-cell infiltration: **(L)** representative sections (scale bars: 100 µm and 20 µm) and **(M)** quantification per field (n = 3). * indicates p < 0.05; *** indicates p < 0.001.

To evaluate therapeutic efficacy *in vivo*, we established a subcutaneous xenograft model using SKOV3-CMAH cells in NXG mice. On day 3 post-tumor inoculation, mice were treated with mGM3/CD28H, hGM3/CD28H, or mock-transduced T cells (**Figure 3A**). Tumor growth was monitored via bioluminescent imaging (BLI). Both CAR-T treatments significantly delayed tumor progression compared to mock controls; notably, hGM3/CD28H-treated mice showed superior tumor regression and growth control (**Figures 3B, C** and **Supplementary Figure S4A, B**). Body weight remained stable in all groups, suggesting minimal systemic toxicity (**Figure 3D**).

To explore the mechanism underlying improved *in vivo* activity of hGM3/CD28H cells, we performed peripheral blood analysis and tumor immune profiling (**Figure 3E**). Flow cytometry revealed significantly higher circulating CD3+ and CAR+ T cells in hGM3/CD28H-treated mice, indicating enhanced *in vivo* expansion or persistence (**Figures 3F, G**). Analysis of tumor-infiltrating lymphocytes (TILs) showed greater infiltration of total CD3+ and CAR+ T cells, as well as increased frequencies of CD137+ and CD107a+ activated subpopulations in the hGM3/CD28H group (**Figures 3H–K**). These findings were corroborated by IHC staining, which demonstrated increased CD3+ cell density in tumor sections from hGM3/CD28H-treated mice (**Figures 3L, M**).

Together, these results confirm that humanized GM3-CAR-T cells possess superior expansion, tumor infiltration, and antitumor efficacy *in vivo*, supporting their translational potential.

### Humanized CD28H-based GM3-CAR-T cells resist functional decline under repeated antigen stimulation, potentially due to reduced tonic signaling

**Figure 4:**
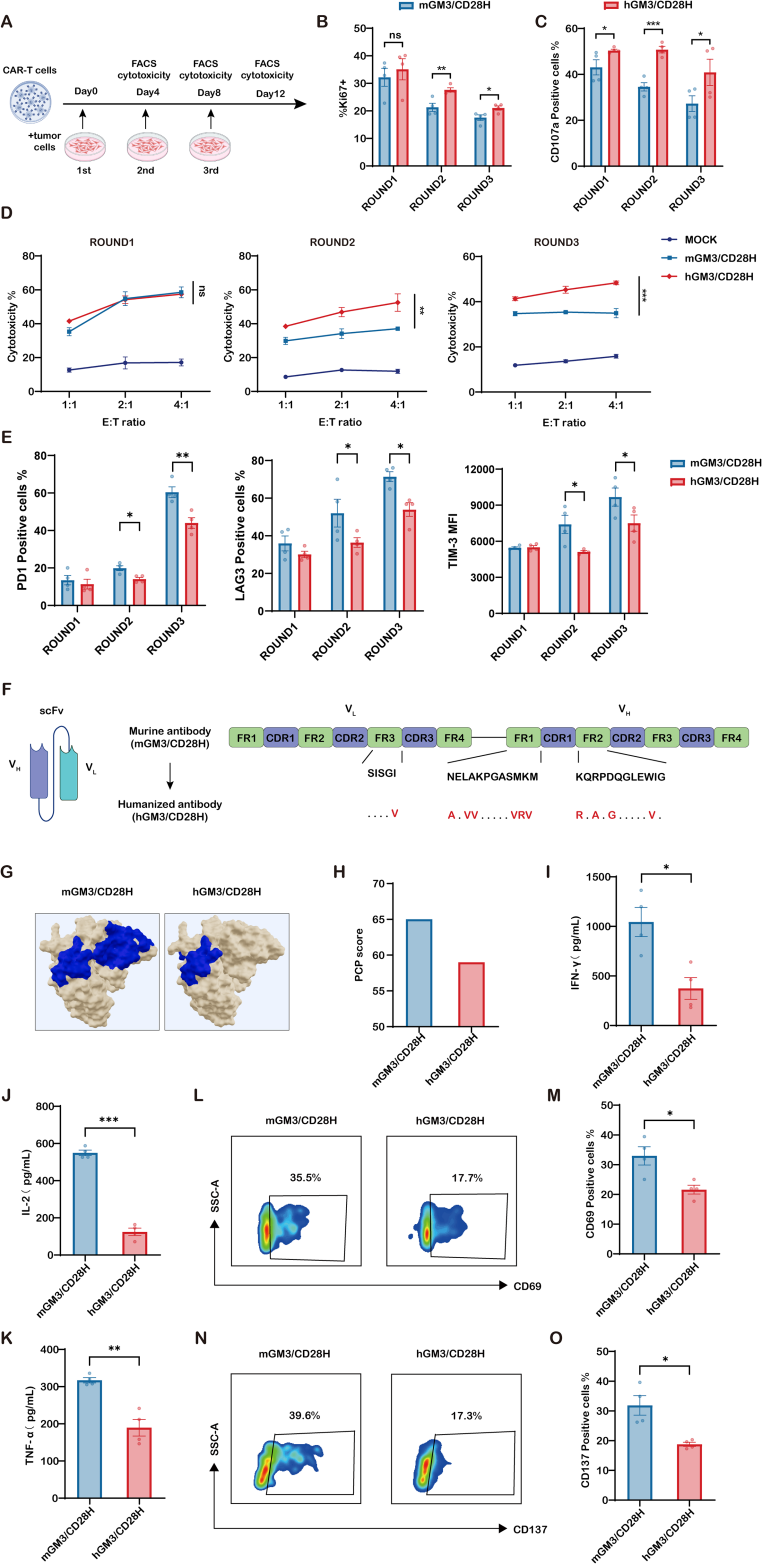
Humanized CD28H-based GM3 CAR-T cells retain functionality under chronic antigen stimulation via reduced tonic signaling. **(A)** Schematic of the repeated stimulation assay. CAR-T cells were co-cultured with SKOV3-CMAH tumor cells for 4 days, then harvested and re-seeded with fresh SKOV3-CMAH cells to initiate the next stimulation cycle. This process was repeated for a total of three rounds. After each cycle, CAR-T cells were collected and analyzed for cytotoxicity, degranulation, and proliferation. **(B)** Quantification of Ki67^+^ CAR-T cells (%) across stimulation rounds, indicating proliferative activity (n = 4). **(C)** Quantification of CD107a^+^ CAR-T cells (%) following 4-hour co-culture with SKOV3-CMAH tumor cells after each stimulation round, indicating degranulation capacity under repeated antigen exposure (n = 4). **(D)** Quantification of tumor cell lysis by CAR-T cells after each round of stimulation. CAR-T cells harvested from each stimulation cycle were re-cultured with fresh SKOV3-CMAH tumor cells at effector-to-target (E:T) ratios of 1:1, 2:1, and 4:1 for 12 hours to assess cytotoxic function (n = 3). **(E)** Expression of exhaustion markers (PD-1, LAG-3, and TIM-3; n = 4).**(F)** The murine anti-GM3(Neu5Gc) scFv 14F7 was humanized to generate 14F7hT by fully preserving all complementarity-determining regions (CDRs) while selectively substituting a limited number of framework residues with human germline amino acids. As illustrated, one substitution was introduced in V_L-FR3, while six and four residues were replaced in V_H-FR1 and V_H-FR2, respectively (red). **(G)** Positive-charge patches (PCP) were displayed by the BindUP web server tool. Beige: solvent-exposed surface; blue: contiguous cationic patches implicated in antigen-independent clustering. **(H)** PCP scores for mGM3/CD28H and hGM3/CD28H CARs, calculated using the CAR-Toner algorithm. **(I–K)** CAR-T cells were cultured for 3 days in the absence of exogenous cytokines and anti-CD3/CD28 stimulation. Supernatants were collected and analyzed for spontaneous secretion of **(I)** IFN-γ, **(J)** IL-2, and **(K)** TNF-α (n = 4). **(L–O)** After 3 days of cytokine and CD3/CD28 withdrawal, CAR-T cells were assessed for surface expression of **(L, N)** CD69 and CD137 (representative flow cytometry plots), and corresponding quantification of **(M)** CD69^+^ and () CD137^+^ cells (n = 4). * indicates p < 0.05; ** indicates p < 0.01; *** indicates p < 0.001; ns, not significant.

Given the superior *in-vivo* performance of hGM3/CD28H CAR-T cells, we hypothesized that they would better withstand functional decline under chronic antigen exposure. We therefore established a repeated-stimulation model (three consecutive 4-day cycles of co-culture with SKOV3-CMAH; **Figure 4A**) and profiled proliferative activity, degranulation, cytotoxicity, and exhaustion. Ki67 analysis showed that the two constructs were comparable after the first cycle, whereas hGM3/CD28H displayed higher Ki67 positivity after the second and third cycles (**Figure 4B**). Concordantly, CD107a degranulation was consistently greater in hGM3/CD28H across cycles (**Figure 4C**). Functionally, the constructs exhibited similar tumor killing after the first round, but a divergence emerged upon repeated exposure: hGM3/CD28H retained potent cytotoxicity through rounds two and three, while mGM3/CD28H showed a progressive decline across E:T ratios (**Figure 4D**). In parallel, exhaustion-associated readouts were attenuated in hGM3/CD28H: compared with mGM3/CD28H, it showed lower frequencies of PD-1^+^ and LAG-3^+^ cells and reduced TIM-3 MFI, particularly after the later cycles (**Figure 4E**). Collectively, these data indicate that hGM3/CD28H sustains proliferation and effector function and exhibits a less exhausted phenotype under repeated antigen stimulation. To explore potential mechanisms underlying the improved functional durability of hGM3/CD28H CAR-T cells, we hypothesized that differential tonic signaling might be involved. As shown in the structural schematic, the murine anti-GM3(Neu5Gc) scFv 14F7 was humanized to generate 14F7hT by fully preserving all complementarity-determining regions (CDRs) while selectively substituting a limited number of framework residues with human germline amino acids—specifically, one substitution in V_L-FR3, and six and four substitutions in V_H-FR1 and V_H-FR2, respectively (**Figure 4F**). Structural modeling using the BindUP server (17) further revealed extensive positively charged surface patches on the extracellular domain of the mGM3/CD28H CAR, indicative of higher antigen-independent clustering propensity. In contrast, the hGM3/CD28H CAR exhibited markedly reduced cationic surface regions (**Figure 4G**). Consistently, the CAR-Toner algorithm (18) predicted a lower tonic signaling potential for hGM3/CD28H CARs compared to mGM3/CD28H CARs. Specifically, the murine construct yielded a positive-charge patch (PCP) score of 65, well above the empirically defined optimal range of 46–56 for supporting T cell fitness (19), whereas the humanized construct scored 59—closer to the ideal threshold (**Figure 4H**). These results suggest that selective framework humanization attenuates tonic signaling by reducing surface charge clustering, thereby contributing to enhanced CAR-T cell persistence and function.

Under conditions devoid of cytokines and antigens, mGM3/CD28H CAR-T cells demonstrated a higher basal secretion of cytokines (IFN-γ, IL-2, TNF-α; **Figures 4I–K**) and an increased expression of activation markers CD69 and CD137 (**Figures 4L–O**). In contrast, hGM3/CD28H cells exhibited a more quiescent phenotype, with CAR-Toner providing directionally concordant predictions (64 vs 59 for mGM3/CD28H and hGM3/CD28H, respectively). In summary, these findings suggest that reduced tonic signaling may contribute to the enhanced proliferative fitness and sustained effector function of hGM3/CD28H. Together, these findings suggest that reduced tonic signaling in humanized CARs may underlie their enhanced proliferative fitness and resistance to functional decline under persistent antigen stimulation, providing a mechanistic explanation for their superior antitumor efficacy *in vivo*.

## Discussion

Tumor-associated glycans such as GM3(Neu5Gc) represent a unique class of targets for CAR-T cell therapy due to their metabolic origin and tumor-selective expression (6). In this study, we systematically engineered and evaluated GM3(Neu5Gc)-specific CAR-T cells using murine and humanized scFv formats, and different hinge domains. Our results demonstrate that both the CD28-based hinge and the humanized scFv framework independently enhance CAR-T cell antitumor activity, persistence, and intratumoral infiltration, culminating in an optimized CAR configuration that combines these two features.

The enhancement observed with the CD28 hinge is likely attributable to its ability to optimize the spatial configuration of the CAR, potentially facilitating more efficient immune synapse formation and receptor clustering (13, 20). These structural features may promote more robust antigen engagement and downstream signaling (21). While our data clearly support the superiority of the CD28 hinge in the context of GM3(Neu5Gc)-targeting CARs, hinge efficacy is known to be influenced by multiple factors, including antigen structure, epitope accessibility, and overall CAR architecture (12–14, 22). Thus, the optimal hinge may vary across different targets, and the favorable performance of the CD28 hinge in this study should be interpreted within the specific design and antigenic context of our CAR system.

In parallel, we found that humanization of the 14F7 scFv reduced tonic signaling and was associated with greater early *in-vivo* accumulation of CAR^+^ T cells (days 6–8) and improved antitumor activity. While consistent with better proliferative fitness under prolonged stimulation *in vitro*, definitive *in-vivo* persistence was not directly assessed and will require longer-term longitudinal tracking. The murine and humanized scFvs shared identical CDR sequences and exhibited comparable antigen-binding affinities, indicating that framework differences—not antigen binding per se—accounted for the altered basal signaling (16). This suggests that antibody humanization may not only reduce immunogenicity (23), as is conventionally appreciated, but also reshape CAR signaling properties by modulating scFv conformation or membrane interactions. Given the potential impact of tonic signaling on T cell proliferative fitness and therapeutic durability (24, 25), this finding underscores the need to consider scFv framework design as a functional parameter in CAR optimization.

To study GM3(Neu5Gc)-targeting CAR-T cells *in vivo*, we employed engineered SKOV3 tumor cells expressing the CMAH enzyme, which restores Neu5Gc biosynthesis and GM3(Neu5Gc) presentation (15). While this model offers a consistent and tunable platform for preclinical evaluation, it does not fully capture the complex and heterogeneous nature of GM3(Neu5Gc) expression in human tumors (15). In exploratory efforts, we attempted to establish patient-derived organoids and xenografts from GM3(Neu5Gc)–positive tumors; however, detectable GM3(Neu5Gc) expression was lost in these models (unpublished observations). This discrepancy likely reflects the dependence of Neu5Gc incorporation on microenvironmental cues and metabolite availability—including stromal interactions and dietary Neu5Gc—conditions that are difficult to reproduce *in vitro* or in immunodeficient hosts. Against this backdrop, we used the SKOV3-CMAH model to provide stable, reproducible GM3(Neu5Gc) expression, allowing us to isolate the effects of hinge selection and scFv humanization on CAR performance. We do not claim that this model recapitulates the full spectrum of antigen densities seen in patients. Indeed, GM3(Neu5Gc) density is heterogeneous across tumors and regions and may evolve under selective pressure. Moving forward, we plan to benchmark activity across patient-derived models with quantified surface density (e.g., antibody-binding-capacity calibration beads) to define efficacy thresholds and inform patient selection, and to explore culture/host modifications that better preserve Neu5Gc availability. Importantly, it is well-established that GM3(Neu5Gc) accumulates selectively in malignant, but not normal, human tissues, even in the presence of systemic dietary Neu5Gc exposure (6, 7). Although the mechanisms underlying this tumor-restricted incorporation are not fully elucidated, cancer cells may exhibit enhanced endocytic activity, metabolic reprogramming, or microenvironment-driven retention of Neu5Gc-containing glycolipids (6). These properties render GM3(Neu5Gc) a metabolically derived neoantigen with high tumor specificity, supporting its candidacy as a safe and effective target for immunotherapy. While humanization can mitigate immunogenicity and, in some contexts, modulate CAR signaling, it also introduces practical constraints. Grafting murine CDRs onto human frameworks typically requires iterative in-silico design and empirical screening, increasing development time and cost (26). Framework substitutions may perturb VH–VL pairing and surface electrostatics, with potential consequences for expression level, aggregation/solubility, and unintended tonic signaling (19). From a manufacturing perspective, humanized binders often necessitate additional characterization (stability, developability, binding comparability) and tighter process controls (27). These trade-offs should be considered when generalizing our findings, and prospective developability and stability assessments should accompany scFv selection in future studies. As an additional limitation, we did not directly profile CAR-proximal or downstream phospho-signaling (CD3ζ, pZAP70, AKT/mTOR, ERK1/2, NF-κB); future work will address this with phospho-flow/Western and single-cell phospho-cytometry.

In conclusion, we identify GM3(Neu5Gc) as a tumor-selective, metabolically defined target for CAR-T cell therapy, and demonstrate that both hinge design and scFv framework engineering play critical roles in optimizing CAR performance. Our data also raise the possibility that antibody humanization may functionally modulate CAR-T cell behavior beyond immunogenicity alone. Future studies should focus on characterizing GM3(Neu5Gc) expression in diverse tumor types, exploring its stability in patient-derived models, and assessing the safety and efficacy of GM3-targeting CAR-T cells in clinical trials.

## Materials and methods

### Cell culture

The human ovarian carcinoma cell line SKOV3 was obtained from the American Type Culture Collection (ATCC) and maintained in McCoy’s 5A Modified Medium (1×) supplemented with 15% fetal bovine serum (FBS; Sigma-Aldrich) and L-glutamine (Sigma-Aldrich). To establish a GM3(Neu5Gc)-positive tumor model for CAR-T cell functional evaluation, SKOV3 cells were transduced with lentiviral particles encoding the murine CMP-N-acetylneuraminic acid hydroxylase (CMAH) gene to generate a stable SKOV3-CMAH cell line. Due to the lack of endogenous CMAH activity, the parental SKOV3 cells do not express GM3(Neu5Gc) under standard culture conditions. For *in vivo* bioluminescence imaging, SKOV3-CMAH cells were further transduced with a lentiviral vector co-expressing GFP and firefly luciferase, resulting in the SKOV3-CMAH-Luc cell line.

Primary human T cells were isolated from the peripheral blood of healthy donors following informed consent. T cells were cultured in X-VIVO 15 medium (Lonza) supplemented with 50ng/mL anti-CD3 antibody (ACRO Biosystems), 1μg/mL anti-CD28 antibody (TLBiotechnology), and 5ng/mL each of IL-2, IL-7, and IL-15 (PeproTech) for 72 hours prior to downstream applications. All cells were maintained at 37°C in a humidified incubator with 5% CO_2_.

### Generation of GM3(Neu5Gc)-targeting CAR constructs

CAR constructs targeting GM3(Neu5Gc) were based on a second-generation lentiviral backbone, comprising an antigen-specific single-chain variable fragment (scFv), a hinge domain, a transmembrane domain, and intracellular signaling domains. Two scFv variants were employed: the murine monoclonal antibody 14F7 and its humanized derivative 14F7hT. The humanized 14F7hT scFv retained the complementarity-determining regions (CDRs) of 14F7, while the framework regions (FRs) were substituted with human germline sequences to reduce immunogenicity while preserving antigen specificity, as previously described (15).

For each scFv, three CAR constructs were generated with different hinge domains: CD8α hinge (CD8H), CD28 hinge (CD28H), or IgG4 hinge (IgGH), resulting in six CAR variants: mGM3/CD8H, mGM3/CD28H, mGM3/IgGH, and their humanized counterparts hGM3/CD8H, hGM3/CD28H, and hGM3/IgGH. All constructs were cloned into the pCDH lentiviral vector and sequence-verified, as reported previously (28).

### Lentiviral packaging and transduction of human T cells

Lentiviral particles were produced by co-transfecting 293FT cells with lentivectors and packaging/envelope plasmids using polyethyleneimine (PEI; Polysciences), following the manufacturer’s instructions. Lentiviral particles were produced by co-transfecting 293FT cells with lentivector and packaging/envelope plasmids using PEI. Supernatants were collected at 48 and 72 h, passed through a 0.45 μm filter, and concentrated by ultracentrifugation, as previously described (29). Activated human T cells were transduced with lentivirus in the presence of 8μg/mL polybrene (Sigma-Aldrich), as previously delineated (30). Transduction efficiency was determined 72 hours post-transduction by flow cytometry using anti-FLAG-FITC antibody (BD Biosciences), which detects the FLAG epitope incorporated into the CAR extracellular domain.

### Flow cytometry analysis

#### T-cell staining

Single-cell suspensions were prepared and washed with cold phosphate-buffered saline (PBS) supplemented with 1% bovine serum albumin (BSA). T-cell surface markers were stained using the following fluorochrome-conjugated antibodies: anti-CD3-BUV395, anti-FLAG-FITC (to determine transduction efficiency via detection of the FLAG tag in the CAR extracellular domain), anti-CD69-APC, anti-CD137-BV421, and anti-CD107a-PE (all from BD Biosciences). Cells were incubated with antibodies for 30 minutes at 4°C in the dark. For intracellular detection of the proliferation marker Ki67, cells were fixed and permeabilized using Fixation/Permeabilization Buffer (BD Biosciences) for 20 minutes at room temperature, followed by staining with anti-Ki67-AF647 for 30 minutes at 4°C. Data acquisition was performed on a FACS Aria II (BD Biosciences), and results were analyzed using FlowJo v10 software.

#### Tumor cell staining

To evaluate GM3(Neu5Gc) expression, tumor cells were incubated with a chicken anti-Neu5Gc antiserum (BioLegend, San Diego, CA) in binding buffer supplied by the manufacturer for 1 hour on ice. After washing with cold PBS, cells were stained with an FITC-conjugated goat anti-chicken IgY secondary antibody (BioLegend). A chicken polyclonal IgY (BioLegend) was used as an isotype control. Samples were analyzed on a flow cytometer as described above.

### Cytokine detection by ELISA

CAR-T effector cells (2.5×10_5_) were co-cultured with target tumor cells (5×10_4_) in 96-well plates for 24 hours. Supernatants were collected, and the concentrations of IFN-γ, IL-2, and TNF-α were measured using commercial ELISA kits (R&D Systems) according to the manufacturer’s protocols.

### Cytotoxicity assay using CFSE/PI labeling

Target cells were labeled with 5μM CFSE (BD Biosciences) at 37°C for 15 minutes, then washed and co-cultured with CAR-T cells at effector-to-target (E:T) ratios ranging from 1:1 to 8:1 for 12 hours. After co-culture, cells were stained with propidium iodide (PI; BD Biosciences) for 15 minutes at room temperature. The percentage of dead target cells (CFSE^+^/PI^+^) was quantified using a BD Accuri C6 flow cytometer and analyzed with FlowJo software.

### Xenograft mouse model

All animal experiments were approved by the Animal Ethics Committee of Beijing Cancer Hospital (Beijing, China) and conducted in accordance with institutional guidelines. Female immunodeficient NXG mice (6–8 weeks old) were purchased from HFK Bio-Technology and maintained under specific pathogen-free (SPF) conditions.

To establish subcutaneous tumor xenografts, 2.5×10^6^ SKOV3-CMAH-Luc cells were injected into the right flank of each mouse. Three days later, mice were randomized into treatment groups based on comparable tumor bioluminescence intensity and received 7×10^6^ T cells via intravenous injection: mock-transduced T cells, mGM3/CD28H CAR-T cells, or hGM3/CD28H CAR-T cells. Tumor progression was monitored at indicated time points by bioluminescence imaging (BLI).

For T-cell trafficking and infiltration analysis, mice bearing SKOV3-CMAH tumors (~100 mm³) were intravenously infused with mock or CAR-T cells. Peripheral blood was collected and subjected to red blood cell lysis, followed by flow cytometry to assess circulating CAR-T cells. On day 8 post-infusion, tumors were harvested, enzymatically digested into single-cell suspensions, and analyzed by flow cytometry to evaluate T-cell infiltration and activation. For the infiltration cohort (n = 3 mice per group), each excised tumor was divided within the same animal: one portion was minced and enzymatically dissociated for flow-cytometric analysis, while the remaining portion was fixed in 10% neutral-buffered formalin (72 h) and processed for paraffin embedding. This split-tumor design provided paired flow-cytometry and histology readouts from the same tumors, minimizing inter-animal variability. On day 6 post-infusion, Peripheral blood was collected, red blood cells were lysed, and leukocytes were stained for flow cytometry with an anti-human CD3 monoclonal antibody (CD3-BUV395, BD Biosciences); where indicated, anti-FLAG-FITC was included to enumerate CAR^+^ subsets. On day 8 post-infusion, tumors were dissociated into single-cell suspensions and analyzed by flow cytometry using an anti-human CD3 monoclonal antibody (CD3-BUV395, BD Biosciences); where indicated, cells were co-stained with anti-FLAG-FITC, anti-CD137-BV421, and anti-CD107a-PE to assess CAR expression, activation, and degranulation.

### Immunohistochemistry

For histology, the formalin-fixed fraction of the Day-8 tumors described above was processed through graded ethanol and xylene for paraffin embedding (10% neutral-buffered formalin, 72 h). Sections (5 μm) were deparaffinized, rehydrated, and stained using a commercial IHC kit (ZSGB-BIO) per the manufacturer’s instructions.

### Computational analysis

Single-chain variable fragment (scFv) structures of mGM3/CD28H and hGM3/CD28H were generated by homology modeling using the ABodyBuilder online tool (SAbPred). Resulting PDB files were subsequently submitted to the BindUP web server to visualize surface-exposed positive-charge patches (PCPs). PCP scores, indicative of tonic signaling propensity, were calculated using the CAR-Toner algorithm based on predicted scFv structures. Higher PCP scores reflect increased tonic signaling potential, associated with greater antigen-independent CAR clustering.

### Statistical analysis

All statistical analyses were conducted using GraphPad Prism 9.5.0. Flow cytometry data were processed in FlowJo v10, and final figures were assembled using Adobe Illustrator 2020. Data are presented as mean ± standard error of the mean (SEM) from at least three independent experiments unless otherwise specified. Comparisons between two groups were analyzed using an unpaired two-tailed Student’s t-test, while comparisons among multiple groups were evaluated using one-way or two-way ANOVA followed by Tukey’s *post hoc* test. The Mann–Whitney U test was used for nonparametric comparisons. Statistical significance was defined as follows: ns (not significant), *P<0.05, **P<0.01, ***P<0.001.

## Data Availability

The original contributions presented in the study are included in the article/**Supplementary Material**. Further inquiries can be directed to the corresponding author/s.
