# Supplementary material for: Framework humanization enhances GM3(Neu5Gc)-targeting CAR-T cell function by reducing tonic signaling
Source: Front Immunol. 2025 Oct 23;16:1697732. doi: 10.3389/fimmu.2025.1697732 (PMC12589110; doi:10.3389/fimmu.2025.1697732)
Supplement: Supplementary file 1 [file DataSheet1.docx]

Supplementary Materials for

**Framework humanization enhances GM3(Neu5Gc)-targeting CAR-T cell function by reducing tonic signaling**

Jiaxin Tu *et al.*

*Corresponding author. Email: chaotingzhang@bjmu.edu.cn and luzheming@bjmu.edu.cn

**This PDF file includes:**

Figures. S1 to S4


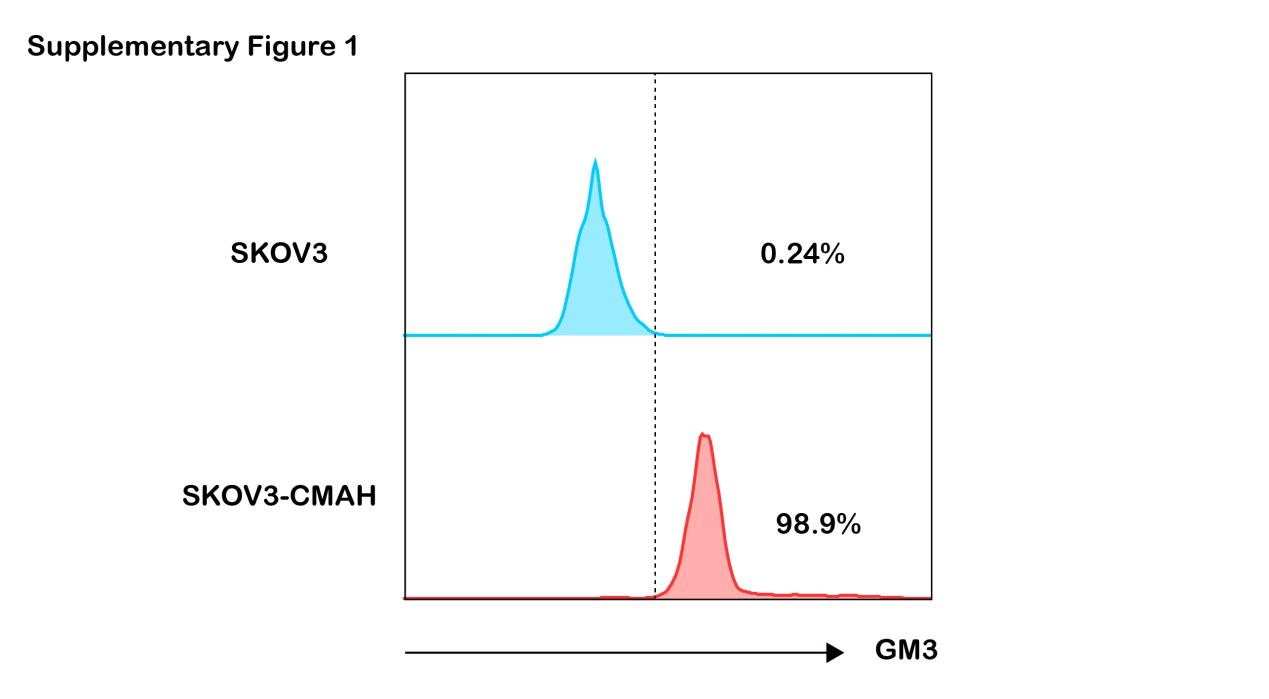


Figure S1. Generation and validation of GM3(Neu5Gc)–expressing SKOV3-CMAH cells by flow cytometry.

Parental SKOV3 or SKOV3 cells transduced with murine CMAH (SKOV3-CMAH) were stained using the 14F7 antibody against GM3(Neu5Gc) and analyzed by flow cytometry. Overlaid histograms show the percentage of GM3(Neu5Gc)-positive cells in each line, with a dashed vertical line indicating the positivity gate. Parental SKOV3 cells exhibit negligible GM3(Neu5Gc) staining (0.24 %), whereas SKOV3-CMAH cells demonstrate robust, homogeneous expression (98.9 %).


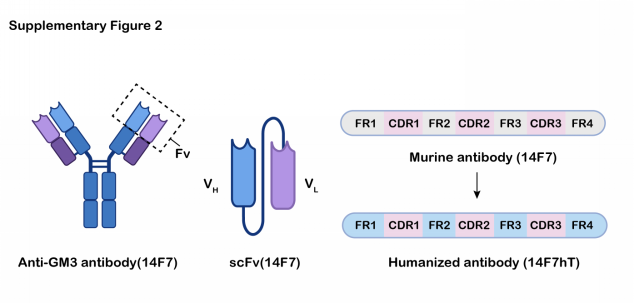


Figure S2. Selective framework humanization of the 14F7 single-chain variable fragment (scFv).

The murine anti-GM3 antibody 14F7 (left) was engineered into a single-chain variable fragment (scFv) by connecting its heavy (VH, blue) and light (VL, violet) chain variable domains with a flexible linker (center). To reduce predicted immunogenicity while retaining antigen specificity, all three murine complementarity-determining regions (CDR1–3, pink) were preserved. The adjacent framework regions (FR1–FR4) were selectively humanized by replacing murine residues with corresponding human germline sequences. Humanized framework regions are shown in light blue, while retained murine frameworks are depicted in grey.


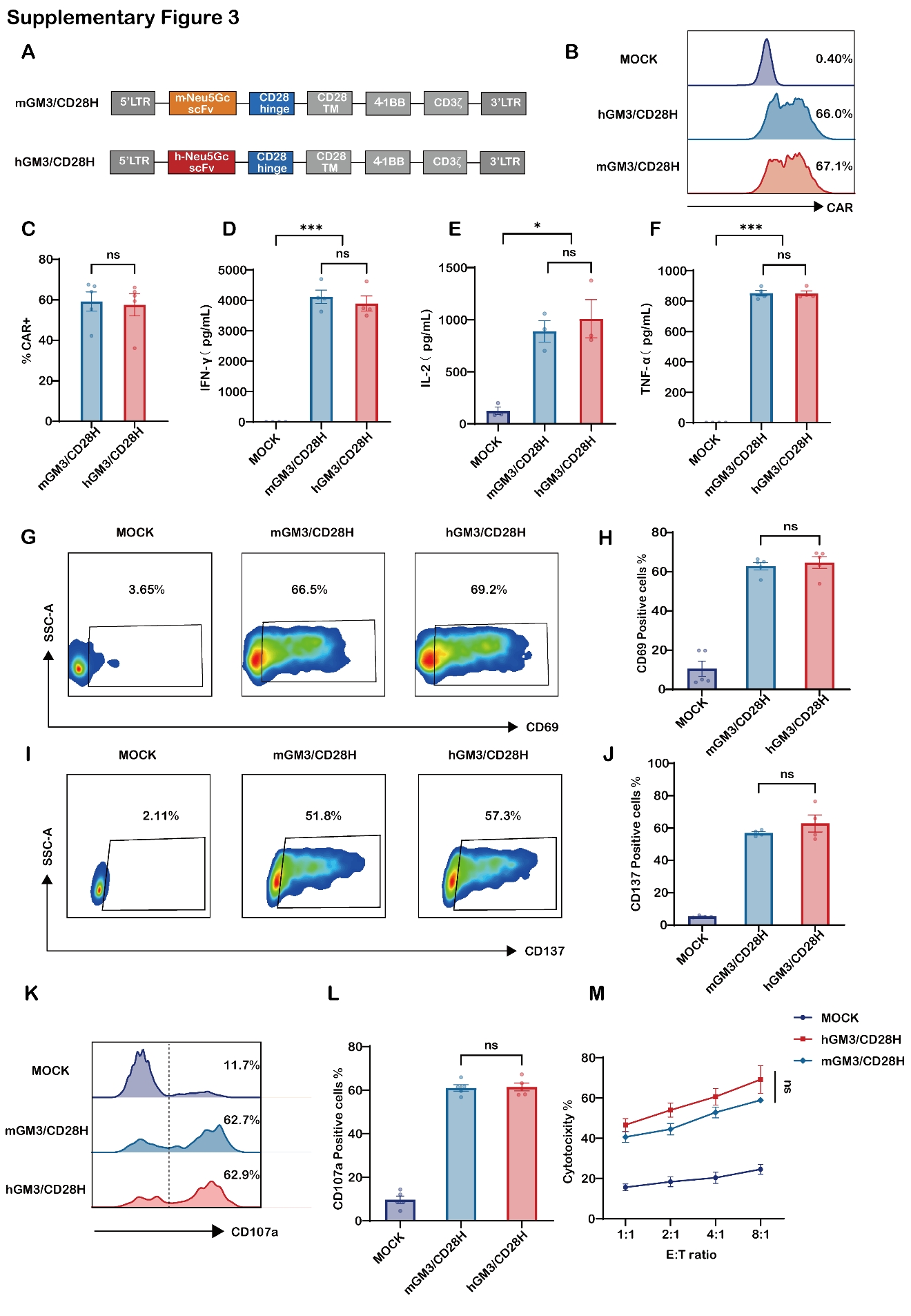


**Figure S3. Murine and humanized CD28-hinged GM3(Neu5Gc)–CAR-T cells exhibit equivalent in vitro functionality.**

(A) Schematic comparison of mGM3/CD28H (murine scFv) and hGM3/CD28H (humanized scFv) constructs with identical hinge and signaling domains. (B) Flow cytometry of CAR expression 72 h post-transduction. (C) CAR⁺ transduction efficiency (n = 5), showing no significant difference. (D–F) Cytokine production after co-culture with SKOV3-CMAH for 24 h (E:T = 5:1): ELISA for (D) IFN-γ (n = 4), (E) IL-2 (n = 3), (F) TNF-α (n = 4). (G–J) Activation marker expression: representative plots for (G) CD69 and (I) CD137, with quantification of (H) CD69⁺ (n = 5) and (J) CD137⁺ cells (n = 4). (K–L) CD107a degranulation assay: (K) representative plots and (L) quantification (n = 5). (M) Cytotoxic activity at various E:T ratios over 12 h (n = 5).


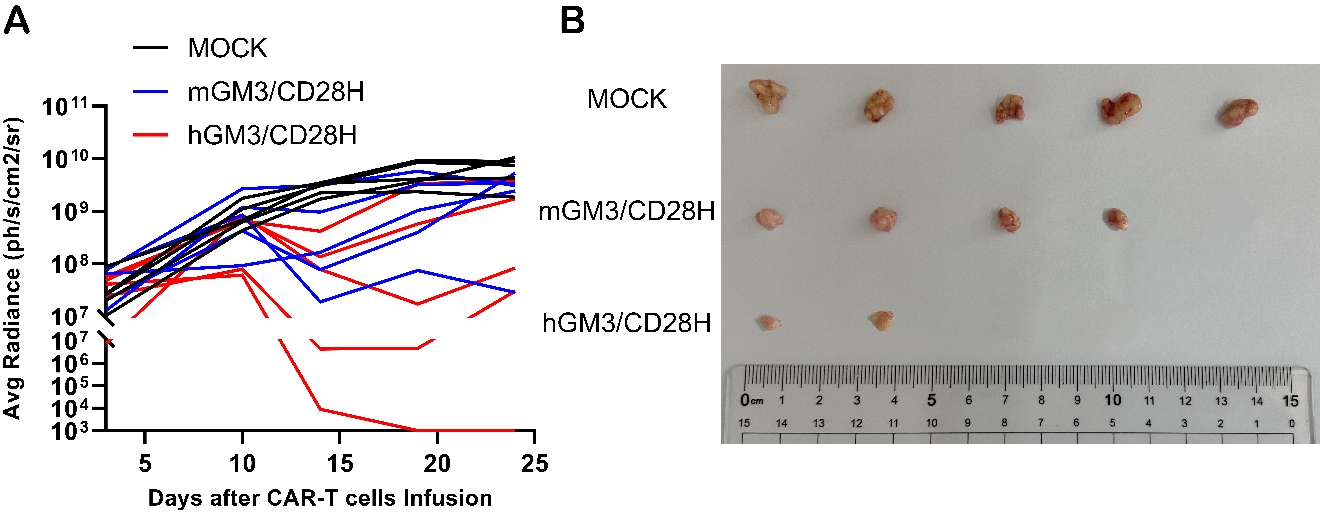


Figure S4. In vivo bioluminescence and ex vivo endpoint tumor readouts.

(A) Individual-mouse bioluminescence trajectories (Avg Radiance, p/s/cm²/sr; log₁₀ scale) plotted over days after CAR-T infusion. Colors denote treatment groups: black, MOCK; blue, mGM3/CD28H; red, hGM3/CD28H. Each line represents one group. The composite overlay is provided to facilitate cross-group visualization while preserving the heterogeneous kinetics observed in vivo.

(B) Representative photographs of excised tumors at endpoint from the three cohorts, arranged by group and shown with a millimeter ruler for scale. The gross tumor sizes qualitatively mirror the imaging trends, with smaller masses seen more frequently in the hGM3/CD28H group.
