# Supplementary figures and images for: Framework humanization enhances GM3(Neu5Gc)-targeting CAR-T cell function by reducing tonic signaling
Source: Front Immunol. 2025 Oct 23;16:1697732. doi: 10.3389/fimmu.2025.1697732 (PMC12589110; doi:10.3389/fimmu.2025.1697732)

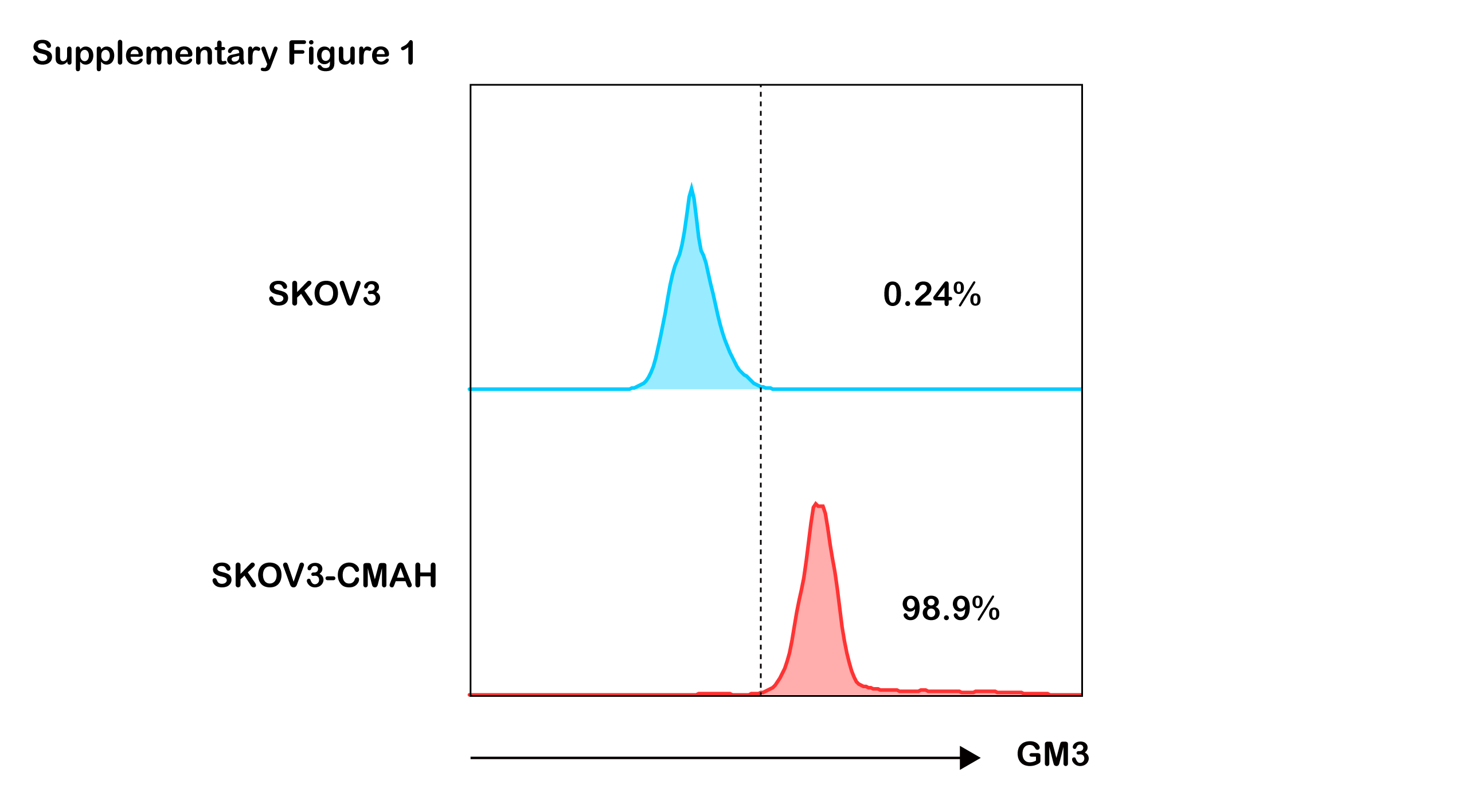

Supplement: Supplementary file 2 [file Image1.tif]

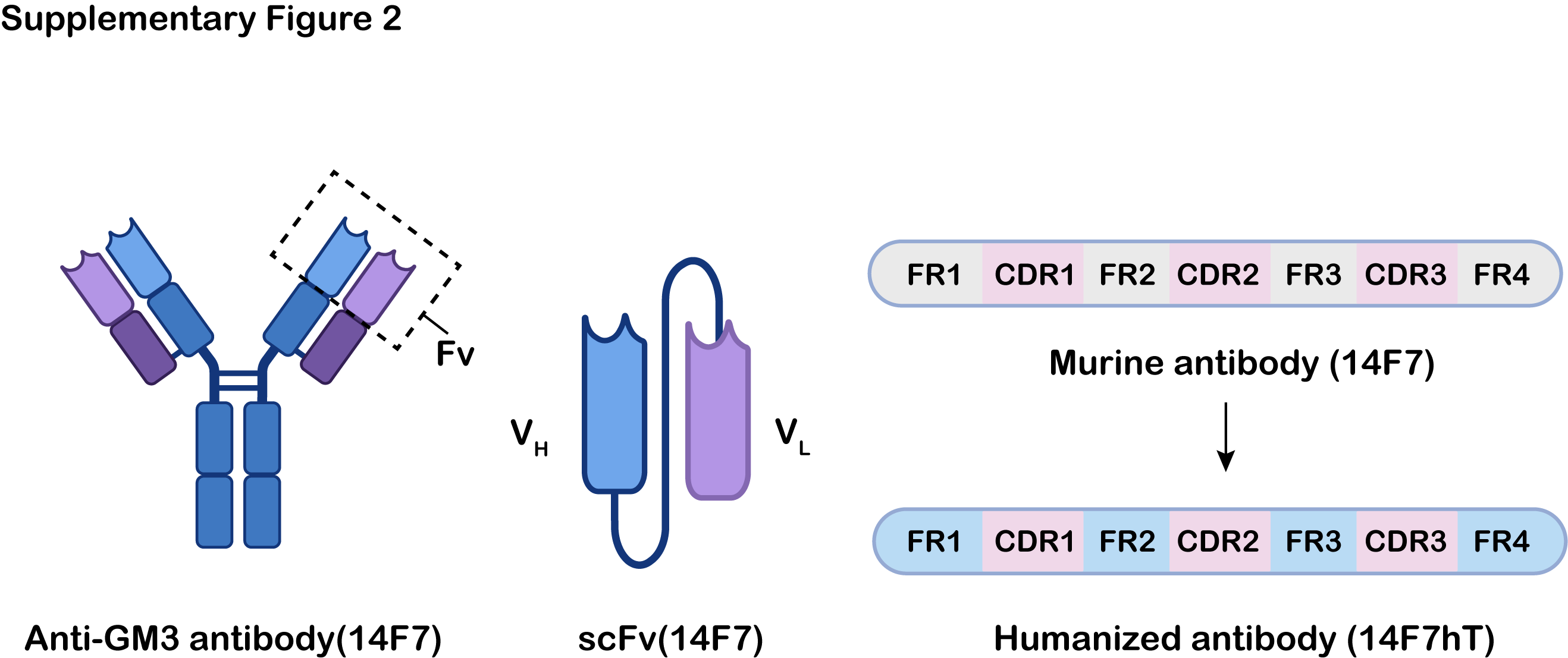

Supplement: Supplementary file 3 [file Image2.tif]

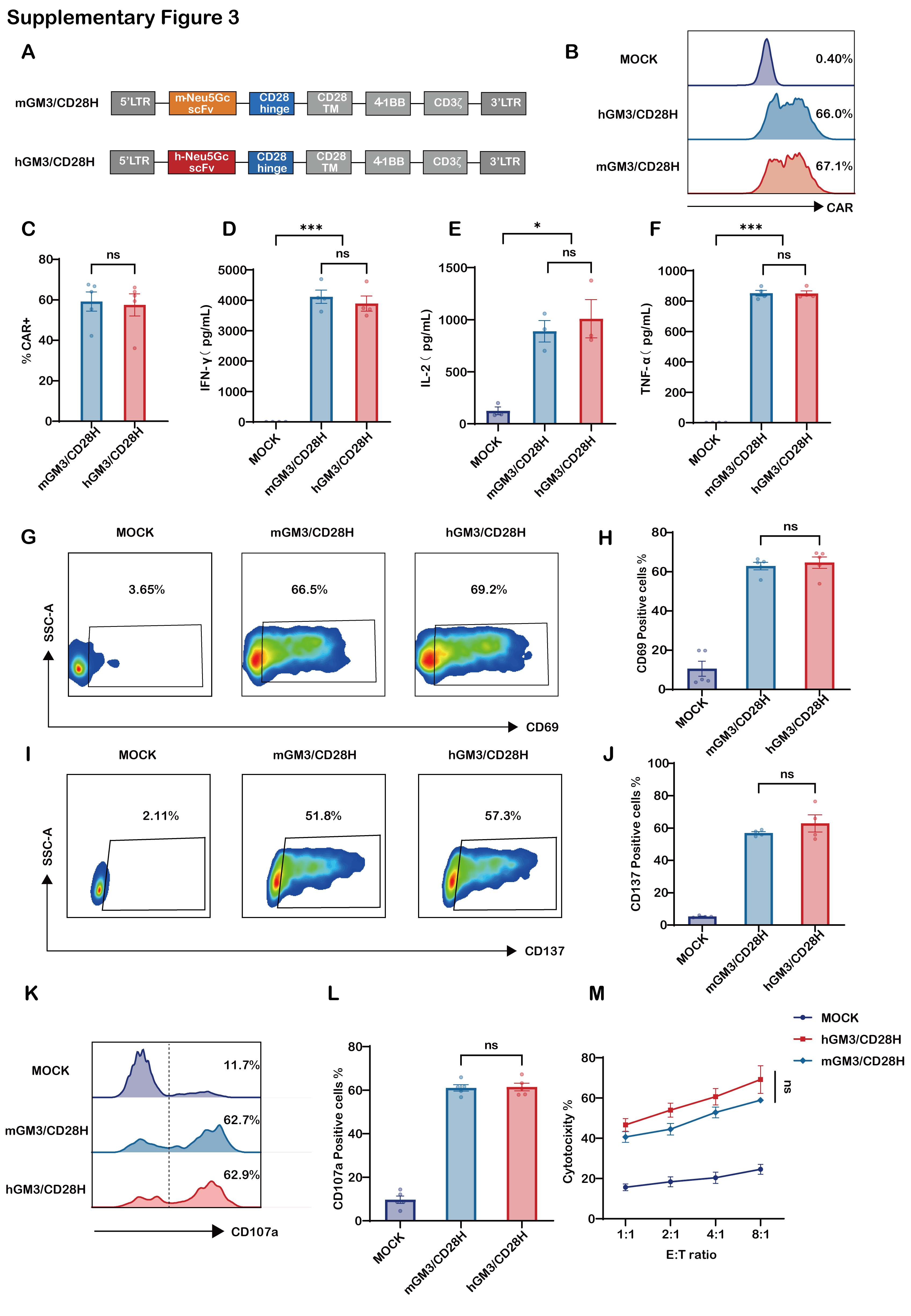

Supplement: Supplementary file 4 [file Image3.tif]

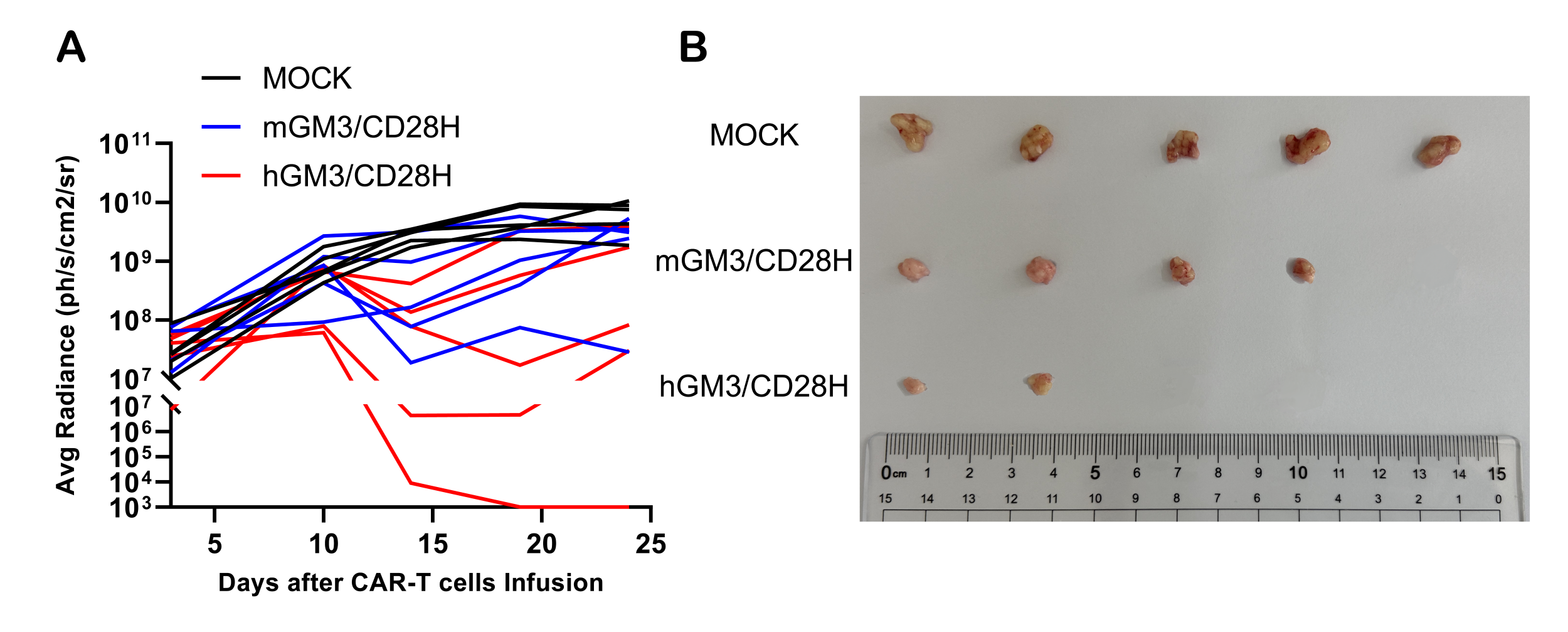

Supplement: Supplementary file 5 [file Image4.tiff]
